# ADRD: A REVIEW OF THE PATIENT-CENTERED OUTCOMES RESEARCH INSTITUTE’S (PCORI) RESEARCH PORTFOLIO AND PRIORITIES

**DOI:** 10.1093/geroni/igad104.3164

**Published:** 2023-12-21

**Authors:** John Audley, Tabassum Majid, Yewande Akinbami, Neeraj Arora, Marissa Coyle, Joanna Philips, Kate Moraras, Steve Clauser

**Affiliations:** Patient Centered Outcomes Research Institute, Washington, District of Columbia, United States; Patient Centered Outcomes Research Institute, Washington, District of Columbia, United States; Patient Centered Outcomes Research Institute, Washington, District of Columbia, United States; Patient Centered Outcomes Research Institute, Washington, District of Columbia, United States; Patient Centered Outcomes Research Institute, Washington, District of Columbia, United States; Patient Centered Outcomes Research Institute, Washington, District of Columbia, United States; Patient Centered Outcomes Research Institute, Washington, District of Columbia, United States; Patient Centered Outcomes Research Institute, Washington, District of Columbia, United States

## Abstract

Given the aging of the U.S. population, the number of individuals living with Alzheimer’s Disease and Related Dementias (ADRD) is projected to reach 13 million by 2050. There is an urgent need for research efforts to advance prevention, diagnosis, and treatment strategies for ADRD. Furthermore, caregivers of individuals with ADRD often face distinct challenges and experience significant caregiving burden that has an impact on their well-being. To address unmet needs of individuals with ADRD and their caregivers, PCORI has invested nearly $85 million in patient-centered comparative clinical effectiveness research (CER) focused on ADRD and has initiated several stakeholder convenings to identify future research priorities. PCORI’s ADRD portfolio of 15 CER studies includes interventions focused on caregiving, polypharmacy, telehealth, care models, and palliative care. Additionally, PCORI has funded engagement awards, dissemination & implementation initiatives, and research infrastructure projects to help facilitate the conduct and awareness of research on persons living with ADRD and their caregivers. To support continued research in this area, PCORI plans to re-issue a $50 million funding initiative on Healthy Aging with a special area of emphasis on interventions for caregivers of older adults living with ADRD. By systematically synthesizing PCORI’s portfolio and themes from stakeholder convenings in ADRD, we can identify the strengths of current investments and understand evidence gaps to guide PCORI’s future investments. This will enable us to holistically address future real-world, decisional dilemmas most important to patients, caregivers, and other stakeholders.

